# The Influence of Processing and the Polymorphism of Lignocellulosic Fillers on the Structure and Properties of Composite Materials—A Review

**DOI:** 10.3390/ma6072747

**Published:** 2013-07-11

**Authors:** Dominik Paukszta, Slawomir Borysiak

**Affiliations:** Institute of Chemical Technology and Engineering, Poznan University of Technology, Sklodowskiej-Curie 1, Poznan 60-965, Poland; E-Mail: dominik.paukszta@put.poznan.pl

**Keywords:** lignocellulosic materials, wood, natural fibres, composites, polymorphs of cellulose, processing and recycling

## Abstract

Cellulose is the most important and the most abundant plant natural polymer. It shows a number of interesting properties including those making it attractive as a filler of composite materials with a thermoplastic polymer matrix. Production of such composite materials, meeting the standards of green technology, has increased from 0.36 million tons in 2007 to 2.33 million tons in 2012. It is predicted that by 2020 their production will reach 3.45 million tons. Production of biocomposites with lignocellulosic components poses many problems that should be addressed. This paper is a review of the lignocellulosic materials currently used as polymer fillers. First, the many factors determining the macroscopic properties of such composites are described, with particular attention paid to the poor interphase adhesion between the polymer matrix and a lignocellulosic filler and to the effects of cellulose occurrence in polymorphic varieties. The phenomenon of cellulose polymorphism is very important from the point of view of controlling the nucleation abilities of the lignocellulosic filler and hence the mechanical properties of composites. Macroscopic properties of green composites depend also on the parameters of processing which determine the magnitude and range of shearing forces. The influence of shearing forces appearing upon processing the supermolecular structure of the polymer matrix is also discussed. An important problem from the viewpoint of ecology is the possibility of composite recycling which should be taken into account at the design stage. The methods for recycling of the composites made of thermoplastic polymers filled with renewable lignocellulosic materials are presented and discussed. This paper is a review prepared on the basis of currently available literature which describes the many aspects of the problems related to the possibility of using lignocellulosic components for production of composites with polymers.

## 1. Introduction—Fundamental Studies on Composites Filled with Lignocellulosic Materials

Renewable cellulosic fibres have been often and increasingly used as reinforcement or as filler of engineer polymers. The composites obtained in this way have gained increasing significance and a wider range of application. Lignocellulosic materials such as wood, natural fibres and fragments of other plants have become an interesting research subject aimed at searching for the optimum materials for specific purposes [1,2,3,4,5,6,7,8]. The interest in lignocellulosic materials stems from their many beneficial features such as renewability, biodegradability, low cost, availability and the ease of their technological preparation. The use of such materials is attractive from both the economic and the environmental point of view. It should be mentioned that the use of renewable materials as composite fillers permits lower use of non-renewable materials making up the polymer matrix. Usually, the polymer matrix is made of polyolefins, but when polymers from renewable sources are used, e.g., PLA, composites are made only of renewable components.

The natural fibres such as sisal, kenaf, hemp and coir have been used often and increasingly instead of glass fibres [9,10,11,12,13], as in general, the specific properties of composites reinforced with natural fibres compare favourably with those reinforced with glass fibres [14]. The composites filled with lignocellulosic materials offer a number of interesting properties of which the most attractive are their excellent mechanical properties, high damping of mechanical vibrations and good absorption of sound waves [15,16,17]. They also have low density which is highly appreciated in the automotive industry.

It is also important that the composites are produced by commonly used methods. The composites are used mainly in the construction industry, automotive industry and for furniture production. Exemplary products include profiles obtained by extrusion, such as pipes reinforced with sisal fibres or doors filled with refined wood, inner lining of car doors, car seat moulds, coatings for damping sound or vibrations, elements of car inside fittings. The main automotive concerns have used composites on a mass scale reinforced with sisal, kenaf, flax or coir fibres.

The most often used lignocellulosic filler is wood from different tree species [18,19,20]. Other frequently used fillers are jute, hemp and flax fibres [21], sisal fibres [22], cotton [23] and abaca [24]. Woody cores of flax and hemp being waste products after extraction of their fibres, have also been used as fillers. There are composites containing mixtures of lignocellulosic materials, for example wood flour with kenaf fibres [25]. Fillers from other plants like e.g., bagasse fibres [26,27], coir [28] or wheat straw [29] have also been used. Recently, much interest has been paid to materials not used in industry as yet, such as rapeseed straw [30,31,32,33].

In recent years interest has grown rapidly in cellulose nanocrystals as fillers of polymers. So far the studies in the area of nanocomposites have concerned mainly inorganic fillers such as nanoclay, montmorilonite, silica and mica, however, recently, the interest in cellulose nanofibril-filled nanocomposites has substantially increased [34,35,36,37,38,39,40,41]. This interest stems from a number of their attractive properties such as renewability, biodegradability, low cost, high availability, high surface area, high aspect ratio, low density, high temperature resistance and high surface reactivity, which permit many chemical modifications [42,43,44]. As indicated by a literature survey [45], cellulose nanocrystals (CN) have been used as fillers of many polymer matrices e.g., polypropylene, poly(vinyl chloride), siloxanes, poly(caprolactone), poly(vinyl acetate), phenol-formaldehyde, and thermoplastic starch. Cellulose nanocrystals have been obtained by breaking down the lignocellulose fillers and isolating the crystalline regions [46]. The preparation of CN involves also the use of sulphuric acid hydrolysis of native cellulose. Acid molecules primarily degrade the less ordered, and thus more accessible regions along the cellulose microfibrils, to finally leave intact nanometric highly crystalline cellulose fragments [47]. CN obtained from wood is of about 100–300 nm in length and 3–15 nm in width [48,49,50]. The properties of the cellulose nanofiller obtained depend on the origin of the lignocellulose filler and the conditions of degradation (time, temperature, pressure) [51,52].

Introduction of CN as a polymer filler brings improvement in the mechanical properties of the nanocomposites obtained [44,51,53,54,55,56]. However, an important limitation on the use of nanofillers is the problem with a sufficient distribution of a nanofiller in the polymer matrix and the high quality of such distribution that is necessary to achieve good mechanical properties of the nanocomposites [48]. Many authors have tackled the problem of nanofiller distribution [57,58]. Yaung [57] found that improved filler dispersion leads to better uniform stress transfer in the composite and more consistent quality of the composite materials. Another problem is the quality of adhesion between cellulose nanocrystals and the hydrophobic polymer matrix. In order to improve the intermolecular interactions, prevent CN aggregation, and improve dispersion of the nanofiller, modification of the cellulose surface has been carried out [59,60,61,62,63].

Besides the improvement of mechanical properties, nanocomposites with cellulose crystals also show barrier properties [45]. They can be applied to the production of membranes for gas separation and water vapour transport. According to literature [45] in the systems of cellulose nanocrystals-hydrophobic polymer matrix the water vapour transport rate (WVTR) increases with increasing content of CN, while in the systems of cellulose nanocrystals-hydrophilic matrix, WVTR decreases with increasing content of CN. Moreover, cellulose nanocrystals act as an active nucleating agent in semicrystalline matrices, which leads to an increased degree of crystallinity of the nanocomposites [64,65]. High nucleation ability of CN has been confirmed by Gray [66], who observed formation of transcrystalline structures of polypropylene in the presence of nanocrystals of cellulose.

On the other hand, the use and production of such composites are marred by some serious limitations. In the process of lignocellulosic materials preparation and in their use, a high water absorptivity must be taken into account, which for some materials exceeds 10%. However, the composites obtained by extrusion, injection moulding or pressing are coated with a layer of the matrix which effectively protects the composite against moisture, e.g., when exposed to the elements. Another limitation is the temperature of processing or reprocessing. The temperature of processing must be adjusted to the resistance of cellulose materials to thermal degradation, which is why polyolefins [3,9,21] and polyvinyl chloride or polystyrene [67,68] are used as the matrix. The first symptoms of thermal decomposition of lignocellulosic materials are alreadyobservable at 140 °C, but it is generally assumed that 200 °C is a safe temperature for processing. Attempts have been made at using other thermoplastic polymers at processing temperatures much exceeding the limiting 200 °C, by assuming a very short contact of the polymer with the lignocellulosic material, but such composites have not found wide application as yet. Another method, although rarely applied, involves the use of resins, for example polyester resins [69]. When resins are used, the problem of filler degradation is irrelevant as the processing temperature is much lower than when using thermoplastic polymers.

In producing composites of reproducible properties a problem related to the inhomogeneity of the substrate may appear, especially when using lignocellulosic materials from annual plants. Other problems may be the time and the different technologies needed to prepare the lignocellulosic materials for the form applicable to the final composite. For instance, in the process of fibre preparation there are operations of fibre separation, and also in rapeseed straw the mechanical size reduction into a proper fraction with removal of the parenchyma tissue.

The most severe problem influencing the functional properties of composites filled with lignocellulosic materials is their insufficient adhesion to the majority of polymers [70,71,72,73]. This problem follows from differences in polarity of the composite components. Lignocellulosic fillers show high polarity related to the chemical structure of cellulose, hemicelluloses and lignin, while the matrices usually are nonpolar polymers such as polyethylene and polypropylene. These differences result in the necessity of application of certain chemical and physical modifications aimed at improvement of the interphase adhesion. Most often, the reactions of hydroxyl groups from cellulose with a variety of low-molecular modifiers are used [74,75,76]. As a result of such a modification, the hydroxyl group is replaced by a group with a much lower polarity and more compatible with the polymer matrix. Most often applied are esterification reactions in which cellulose is subjected to acid anhydrides, acisa or acid chlorides. On an industrial scale, lignocellulosic materials are most often modified with acetic acid anhydride. Other methods of chemical modification are etherification, reactions with organosilane compounds, furfurylation, reactions with isocyanates, reactions with titanium or borate compounds, and treatment with compounds which contain methylol groups [77]. There is also an enzymatic modification that can be applied, although it is mainly used for wood fillers. Another approach to improve adhesion in the system of lignocellulosic material and thermoplastic polymer is to add a compatibilizer to the mixture. Such a promoter of adhesion can be for example maleic anhydride. At elevated temperature a two-step reaction takes place: the first step is the reaction of maleic anhydride with polypropylene and the second step is esterification. An easier procedure is the use of maleated polypropylene MAPP [78] for production of composites with lignocellulosic materials.

There are also a number of physical methods of modification of lignocellulosic materials, including stretching, calendering thermal treatment, production of hybrid yarn and methods based on electric discharge, which do not change the chemical structure of the fillers. In the method of corona treatment the surface of material subjected to it becomes oxidised, the number of polar groups increases and the surface energy changes. This method can be applied to the lignocellulosic materials and to the polymer matrix. Similar effects are achieved by the method of cold plasma. For example, to improve the adhesion between composite components, S. Marais *et al*. [69,70,71,72,73,74,75,76,77,78,79] proposed the cold plasma treatment to activate flax fibres. An interesting method applied for wood fillers is thermal modification of lignocellulosic materials, although it leads to deterioration of the mechanical properties of the composites manifested by the appearance of cracks and splitting.

A highly beneficial but not necessary step is mercerization of lignocellulosic materials prior to proper chemical modification aimed at improvement of their adhesion to the polyolefin matrix. Mercerization improves the mechanical properties of cellulose thanks to the phase transition and also deshields the groups susceptible to chemical modification. The problems concerning mercerization of lignocellulosic fillers in the context of obtaining different crystallographic varieties of cellulose are discussed in Section 2.

## 2. Influence of Polymorph Cellulose on the Structure and Properties of Lignocellulosic/Polymer Composites

Lignocellulosic fillers can differ in their properties and in the contents of components. The components of such fillers can be divided into two groups (a) main or structural ones (cellulose, hemicelluloses and lignin); and (b) subsidiary or non-structural substances (e.g., natural resins, essential oils, vegetable fats and wax, tannins, dyes, and proteins).

The main component of lignocellulosic materials is cellulose and it determines the physicochemical properties of composite materials. The structure and morphology of cellulose have been studied by many authors. Cellulose is a natural polysaccharide built of β-D-glucopyranose residues (glucose) linked by β-glucosidic bonds 1.4. The planes of subsequent glucoside residues are rotated with respect to each other by 180°, which means that the geometrically repeated element of the cellulose chain is made of two neighbouring glucose residues (a cellobiose residue). Because of the presence of polar hydroxyl groups, the macromolecules are interconnected with many intermolecular bonds in the form of hydrogen bridges. In consequence, the energy of molecular cohesion is relatively high, which leads to high stability of the cellulose molecules, high mechanical strength and high resistance to most solvents. The macromolecule of cellulose makes very long chains. In nature, cellulose chains have a degree of polymerization (DP) of approximately 10,000 glucopyranose units in wood cellulose and 15,000 in native cotton cellulose [80]. In general, native celluloses have DP much higher than regenerated cellulose (DP = 200–500) [81]. The supermolecular cellulose occurring in plant fibres is characterised by a high degree of crystallinity. Cellulose can occur in four polymorphic varieties denoted as I, II, III and IV. As generally accepted, the native cellulose I crystallises in the monoclinic system (*a* = 7.9 A, *b* = 8.35 A, *c* = 10.3 A, γ = 96°) [82]. Later other crystallographic models of cellulose were proposed, which were proved to result from different experimental conditions [83,84,85,86,87]. However, development of new experimental methods has enabled the disclosure that cellulose I is comprised of two crystalline structures, labelled as I_α_ and I_β_, whose contents depend on the origin of cellulose [88,89]. According to Sugiyama *et al*. [90] cellulose I_α_ crystallises in a triclinic system, while I_β_ crystallises in a monoclinic one. Both varieties coexist in the same crystalline microfibrils. The crystallographic structure of cellulose was also studied by Kono *et al*. [91] who applied ^13^C NMR and by Nishiyama *et al*. [92,93] who applied synchrotron X-ray and neutron fibre diffraction. Cellulose I_α_ is metastable and can be easily transformed into a more stable I_β_ as a result of annealing in different chemical media [94,95]. Cellulose II is more thermodynamically stable than I. It is formed in the process of mercerization, *i.e*., treatment of cellulose I with solutions of sodium hydroxide. The process of transformation of cellulose I to cellulose II can take place upon treatment with other chemical compounds such as potassium hydroxide and lithium hydroxide [96,97]. Moreover, sodium hydroxide solution is a good choice because it is cheap and does not require any expensive or toxic organic reagents or special procedures. Transformation of cellulose I into II is realised in steps, through alkalicellulose and hydratocellulose [98,99,100,101]. Penetration of NaOH into the spatial lattice of cellulose I jostles the macromolecules leading to characteristic changes in the parameters of the lattice. The next step is washing out of NaOH with water, which leads to further changes in the lattice (hydratocellulose). After removal of water by drying, the crystal lattice of cellulose II is obtained. The elementary cell parameters of cellulose II are *a* = 7.92 A, *b* = 9.08 A, *c* = 10.34 A, γ = 117.3°, after Kolpak and Blackwell [102]. Upon mercerization such components as hemicelluloses, lignin and extractable substances are removed from the lignocellulosic materials [103,104,105,106,107]. A characteristic feature of the transformation of cellulose I to II is decrystallisation accompanied by a decrease in the perpendicular size of the crystallites and a decrease in the perpendicular size of fibrils from about 20 nm to about 5 nm [108,109]. It should be mentioned that the changes in the spatial structure are only partial. The degree of phase conversion of cellulose depends on the concentration of sodium hydroxide, time of modification and temperature of the process. As reported by Zeronian *et al*. [97], below a certain minimum concentration of sodium hydroxide the process of mercerization does not take place. According to some authors [110,111,112,113,114], the transformation from cellulose I to II takes place for sodium hydroxide solutions of concentrations higher than 10%. It should be noted that the degree of this transformation also significantly depends on the type of the lignocellulosic material, for example the minimum concentration of NaOH needed for the cellulose I to II transformation for pine wood was 15% [64,113], for bamboo fibres it was 12% [115], while for beech wood and flax fibres it was 10% [112,113,114]. The effectiveness of cellulose I to II transformation also depends on the type of lignocellulosic material. Mercerization of pine and salix wood [113,114,115,116] gives about 45%–65% of cellulose II, while that of beech wood [112] gives only 37%–52%. In general, mercerization of wood is less efficient than that of other lignocellulosic materials such as flax fibres [114] and bamboo fibres [115] for which the yields of transformation were 72% and 82%, respectively. Very interesting is the mercerization of wooden parts of rapeseed straw, applying a 5% NaOH solution. Already after one minute the yield of transformation to cellulose II is 30%–40%, depending on the variety of rape. For rapeseed straw the highest yield of cellulose I to II transformation of over 50% was obtained applying a 12.5%–20% Na OH solution with the alkalisation process lasting at least 5 min [117]. The character of time dependence of the yields of cellulose I to II transformations for NaOH solutions of different concentrations is also worth considering. It has been reported that the yield of the transformation for pine [113] and beech [112] wood increases with increasing time of mercerization to about 90 min. For flax fibres [114] the content of cellulose II rapidly increases for a short time of mercerization, up to 10 min, and then it remains at the same level. The effect of the alkaline solution is still quicker for cellulose in rapeseed straw, the yield of transformation is already very high after 1 min of treatment with a NaOH solution, it reaches the maximum value after 5 min and then remains unchanged up to 120 min of mercerization [117].

Differences in the character and efficiency of mercerization can follow from different susceptibilities of the lignocellulosic materials to the cellulose I-II transformation, which can have significant influence on the processes of nucleation and crystallisation of the polymer matrix. As follows from literature data [118,119,120], mercerization leads to deterioration in the nucleation activity of lignocellulosic fillers in polymer composites. Many authors [121,122] have explained this by the possibility of epitaxy between the semicrystalline matrix and the cellulose molecule. Identification of the type of nucleation (epitaxial or non-epitaxial) in the systems of isotactic polypropylene and pine wood was the subject of our earlier paper [111]. It was shown that a greater content of cellulose II in pine wood is responsible for decreased nucleation activity, which is manifested by longer half-time of crystallization, lower degree of phase transformation and lower ability in the formation of transcrystalline structures. On the basis of the theory of heterogenic nucleation [123] it has been shown that polymer-wood composites in which the wood contains only cellulose I (unmodified or alkalised under the conditions not inducing cellulose I transformation to cellulose II) show nucleation of epitaxial character, while when the wood contains both polymorphic varieties a tendency towards non-epitaxial nucleation is observed.

Moreover, upon mercerization, the parallel arrangement of cellulose I chains is changed into a more stable unparallel one, typical of cellulose II [98,102,109,124,125]. This change suggests that cellulose II should have better mechanical properties than cellulose I. Joseph *et al*. [126] reported improvement in the mechanical properties of composites after alkalisation of sisal fibres. This observation was explained by increased roughness of the lignocellulosic filler after removal of low-molecular components. Similar conclusions were drawn by Qin [127], who reported that the composites of polypropylene with ramie fibre subjected to mercerization by 9% NaOH had evidently higher tensile strength. Also Kuruvilla *et al*. [11] and Marcovich *et al*. [128] noted that the removal of hemicelluloses, lignin and wax in the process of mercerization leads to increased roughness of the lignocellulosic filler and determines the character of the filler fibrillation. The process of fibrillation results in an increased ratio of the length to diameter of the filler and hence in enlargement of the area of the filler contact with the polymer matrix, which can improve the mechanical properties of the composites. Albano *et al*. [129] reported that lignocellulosic materials show a tendency towards agglomerate formation, which is a consequence of lignin presence. The agglomerates are responsible for a greater size of the filler particles and a decrease in adhesion to the polymer matrix. Upon mercerization lignin is removed, so the probability of agglomerate formation decreases and improves the dispersion of filler particles in the polymer matrix. The same authors also claim that alkalisation contributes to fibrillation of the filler and improves the readiness of the filler surface for interaction with the polymer matrix. The content of lignin and cellulose influences the interphase adhesion between the polymer and lignocellulosic filler as lignin inhibits the diffusion of cellulose particles into the polymer chains. The above phenomena indicate a positive role for mercerization, as a process removing not only fats and impurities but also lignin and hemicelluloses from the surface of the filler, which thus improves the polymer-filler interactions.

Albano *et al*. [129] noted that alkalisation leads to an increase in Young modulus and tensile stress on breaking of PP composites with wood or sisal fibres. Also Kaith *et al*. [130] proved a positive influence of mercerization on the mechanical properties of polymer composites with flax fibres, which they explained by increased roughness of the filler surface and improvement in its mechanical strength as a result of mercerization leading to improved mechanical properties of the composites.

However, many authors [131,132,133,134,135] have not corroborated these results and reported better mechanical properties of the systems containing cellulose I. Ishikawa *et al*. [109] observed a significant decrease in Young modulus (by about 20%) and an increase in the elongation at break (by about 30%) for systems containing cellulose II after mercerization. Pimenta *et al*. [133] proved that the use of a 3% or 10% NaOH solution for mercerization of sisal fibres does not cause significant changes in mechanical strength of the composites, the only considerable change was an increase in impact resistance. A completely different role of low-molecular substances present in the lignocellulosic filler was described by Raj *et al*. [135]. According to these authors, lignin, hemicelluloses and wax are responsible for better adhesion and dispersion of the filler in the polymer matrix. This conclusion suggests that mercerization would lead to deterioration of the mechanical properties of composites.

The above controversy over the influence of mercerization on the mechanical properties of composites with lignocellulosic fillers was discussed in our earlier work [136]. It was established that the degree of improvement in mechanical properties of composites depends on the concentration of the NaOH solution applied. Mercerization with a 10% NaOH solution does not cause significant changes in the mechanical strength of PP-wood composites, which remain similar to those with wood not subjected to any chemical treatment. The use of NaOH solutions of higher concentrations leads to significant deterioration in mechanical properties of PP-wood composites. These observations suggest that the mechanical properties of such composites depend mainly on the content of the polymorphic varieties of cellulose. Mercerization in the conditions not inducing cellulose I to II transformation has no effect on the mechanical properties of the composites.

## 3. Processing and Recycling of Composites Made of Polymers with Lignocellulosic Fillers

The previous chapter concerned the effect of polymorphic varieties of cellulose on the structure and mechanical properties of polymer composites with lignocellulosic fillers. Another factor determining the macroscopic properties of composite systems made of a polymer matrix and lignocellulosic filler is the presence of stress. The stress at the interphase border appears for example upon such processing as extrusion and injection moulding. The stress generated by the flow of the molten polymer matrix or by displacement of filler in the molten polymer can significantly influence the development of transcrystalline structures. Gray [137] noted that movement of carbon fibres in molten polypropylene can lead to the appearance of transcrystalline structures, which was not observed in the systems with stationary filler. Cai *et al*. [138] reported similar finding for the composites with glass fibres, while Thomason *et al*. [139] made such observations for aramid, glass and carbon fibres. Varga and Karger-Kocsis [140] proved that movement of a fibre in a molten polymer matrix leads to formation of an ordered structure called α-row-nuclei near the filler surface. Lamellar growth of the α-row-nuclei layer takes place as a result of epitaxy on the oriented polypropylene chains produced upon the polymer matrix shear by the moving filler. The above authors supposed that the presence of mechanical stress can be responsible for the formation of α-row-nuclei. According to Karger and Varga [140,141] two variants of the interphase structures can appear, a transcrystalline layer and cylindrical structure, corresponding to different mechanisms of crystallisation at the interphase border.

An interesting problem that needs to be considered is the polymorphism of the polymer matrix. Leugering and Kirsch [142] were the first to note that the presence of shearing forces in iPP matrix induces formation of β polypropylene. Then Devaux and Chabet [143] showed that β-PP can also form in the vicinity of a moving fibre at temperatures below 411 K. Thomason *et al*. [139] determined the limiting temperature of crystallisation as 413 K, above which transcrystalline structures were observed to develop only for the monoclinic variety. The value of this temperature was confirmed by Varga *et al*. [140] and Assoulini [144]. A considerable content of β-iPP in the range of crystallisation temperatures 100–140 °C follows from the fact that in this range the growth rate of β-iPP is higher than that of α-iPP [145]. An important parameter affecting the development of cylindrical structure is the temperature at which the filler fibre was moved in the molten polymer matrix. Varga and Karger-Kocsic [140] proved that above the temperature at which the carbon fibre was moved (463–468 K), a cylindrical structure made exclusively of α-iPP was formed.

The problem of isotactic polymorphism of polypropylene in the presence of fibres has been studied by many authors but the mechanism of formation of individual forms and their phase transformation has not yet been fully solved. In our earlier papers [146] we analysed the behaviour of flax and hemp fibres upon displacement in the polymer matrix. The content of β-iPP was found to depend significantly on such technological conditions as the temperature at which the lignocellulosic filler was moved and the rate of its movement. The supermolecular structure of polypropylene composites with natural fibres was also dependent on the temperature of extrusion [147]. It has been established that there is a specific critical temperature of extrusion above which only the α-iPP variety is formed. The supermolecular structure also depends on the content of the lignocellulosic filler. A higher content of flax fibres is responsible for higher shear stress and hence a greater contribution of β-iPP. The authors of [148] analysed the influence of the parameters of extrusion of composites made of polypropylene and pine wood on the supermolecular structure of the polymer matrix and concluded that there was a significant influence of the extrusion mould on the content of the polymorphic varieties of polypropylene. The application of higher rates of cooling favours the formation of β-iPP. Changes in the supermolecular structure of the polymer matrix are explained by the differences in the kinetics of β→α transformation and relaxation processes. Another problem important for the shearing force appearing upon composite processing is related to the necessity of taking into account the chemical treatment of the lignocellulosic fillers. Chemical modification of the fillers changes the topography of their surface. According to the results of our earlier studies [148] the highest shear stress at the polymer-filler interphase border appear in composites with unmodified wood, which leads to formation of prevalent amounts of the hexagonal variety of polypropylene. The lignocellulosic fillers subjected to chemical processing have a smooth surface and induce lower shear stress at the polymer-filler interphase border.

The structure of composites depends considerably on the viscosity of polymer under the conditions of processing, closely related to the melt flow rate (MFR). The structure of polymer matrix was found to be different in different regions of the extrudate or moulding pieces. As we reported in [149] on the basis of investigation of polypropylene composites with native and modified rapeseed straw, a much higher content of β-iPP was obtained for polypropylene of low MFR index. It should be noted that the internal and external moulding piece of such composites showed a high difference in the content of the hexagonal form of polypropylene. For example, using polypropylene of MFR index 25 g/min or 50 g/min, no hexagonal form was observed inside the moulding piece. These observations were explained as a result of differences in the shearing forces generated mainly by the presence of lignocellulosic fillers and differences in the cooling rates. In contrast to the results for wood fillers, it was found that modification of rapeseed straw with acetic acid anhydride leads to increased efficiency of the shearing force, manifested as a small increase in the content of the hexagonal form of polypropylene in the matrix.

It should be emphasised that the hitherto discussed works on composite processing have concerned only the single processed systems. However, recently much attention has been paid to the important question of the possibility of recycling of composites with lignocellulosic fillers. The following part of this section is devoted to this problem.

Recycling of WPC composites permits curbing the use of non-renewable substrates [150], so the possibility of recycling of products should already be considered at the stage of their design and with regard to two main aspects. On the one hand, the possibility of repeated use of materials for production of certain functional objects should be considered, but on the other hand, the possible effects of repeated processing of materials (the repeated effects of temperatures and shearing forces) on the properties of the final products should be checked. The second aspect has by far the greater cognitive value. Possibilities of recycling of thermoplastic polymer composites with lignocellulosic fillers have been studied by many authors who have concentrated mainly on mechanical characterisation, structural and rheological properties and the effect of the filler on crystallisation of the matrix [151,152,153]. From the practical point of view, the most important seems to be the change in mechanical properties of products after subsequent cycles of recycling.

As far as recycling of polyolefin composites with lignocellulosic fillers is concerned, these materials can certainly be subjected to energy reclaim. There is no contraindication against combustion of such materials under controlled conditions, as in incinerating plants. This method should be used when the amount of material is too small to perform a recycling procedure or when the composites have been contaminated.

Because of their specific properties, the composites reinforced with natural fibres are more suitable for typical recycling than those with glass fibres. When the matrix is a type of resin, then the best method of utilisation is particle recycling which involves refinement of the composite, separation of the powder and fibre fractions followed by addition of these fractions to newly produced products. The development of ways of composite reinforcement by natural fibres instead of the hitherto used glass fibres has been prompted by the fact that lignocellulosic fibres are less brittle and less hard, so their properties are more stable upon repeated processing [154]. Mechanical properties of fibres are described by the aspect ratio which is the ratio of the fibre length to its diameter. For natural fibres this ratio decreases upon subsequent cycles of recycling as both the length and the diameter of fibres get reduced.

Beg and Pickering [155] described the material recycling WPC as thermomechanical as at particular stages of this process the material is subjected to elevated temperatures and mechanical methods of refinement. The repeated processing changes the matrix and the lignocellulosic filler, affects the compatibilizer (if used) and changes the character of interaction between the composite components. The processes taking place in the polymer matrix are the same as those taking place in unmodified polymers. The fibres reinforcing the composite are sensitive to high temperature and are shortened as a result of the shearing forces appearing upon recycling [147,154]. In the process of grinding (Betting) the fibres are shortened and undergo internal and external fibrillation [155]. On the other hand, the distribution of lignocellulosic material in the matrix is improved because of more effective mixing of components, which has a beneficial effect after the first few cycles of recycling. However, with increasing number of cycles the contact between the fibres and the polymer deteriorates. Moreover, the compatibilizer added to the composite loses its properties after a few cycles [156].

From the point of view of applications the change in mechanical properties of the composites after subsequent cycles is important. Bourmaud and Baley [156,157] studied the effects of multiple recycling on the properties of polypropylene composites reinforced in 30% with heme and sisal fibres. For the PP/sisal composite a decrease in Young modulus was observed, while the Young modulus of the PP/heme composite remained practically unchanged [156]. The reduction in the aspect ratio for both fibres was insignificant because of a simultaneous reduction in the length and in the diameter of fibres upon processing [157]. The composites subjected to recycling show an increase in elongation at break. The use of polypropylene with maleic anhydride leads to an increase in elongation at break, but after a few cycles its value is reduced as the multiple recycling causes degradation of the compatibilizer which deteriorates the adhesion at the interphase border. The SEM observations confirmed deterioration of the fibres wettability by the matrix and revealed a large number of damaged fibres [156].

Bakkal *et al*. [158] studied the possibility of reinforcement of low density polyethylene composites with cotton fibres and tested the changes in mechanical properties of the composite upon multiple recycling. They analysed the composites containing 12.5% and 25% of fibres. It was shown that tensile strength increased after the first few cycles of reprocesing and then decreased. Deterioration of mechanical properties could be related to gradual damage to fibres and deterioration of the adhesion between the fibres and the matrix.

Beg and Pickering [155,159] subjected polypropylene composites with 40% and 50% contribution of Californian pine flour (*Pinus radiata*) to recycling. They observed improvement in mechanical properties after the first recycling. For the composite containing 50% of the filler, the Young modulus increased after the first recycling and then its value gradually decreased with increasing number of recycling repetitions. Recycling of composites also influences their tensile strength. Composites with natural fibres show a decrease in tensile strength as the fibres undergo shortening and damage upon repeated recycling and their ability to reinforce polymers wanes. Moreover, an increase in elongation at break was noted with an increasing number of recycling repetitions. This increase could result from the decreasing number of micropores present in the composite, leading to increasing density of the composite.

The subject of our study [160] was recycling of polymer composites with 30% of rapeseed straw, either native or subjected to mercerization followed by modification with acetic acid anhydride. We have found that multiple recycling did not result in deterioration of the mechanical properties of the composites studied. As a result of multiple recycling, the elongation at break of a moulding piece increased but no changes were noted in the tensile strength and Young modulus of the composites and the changes in their impact resistance and hardness were insignificant. It should also be mentioned that no significant differences were observed in the properties of the composites with native rapeseed straw and those with the straw modified with acetic anhydride.

## 4. Conclusions

As illustrated by the results presented in this paper and reported from many renowned research centres, lignocellulosic materials have been often and increasingly applied as fillers of polymer composites. They are safe in the phases of preparation for production, production and recycling, and their properties can be controlled and adjusted to specific needs. Production of composites of very good macroscopic properties is not easy and many aspects need to be considered. One of the most important problems is insufficient adhesion between the phases of polymer matrix and lignocellulosic filler, which has been solved hitherto by different types of chemical or physical modifications of the composite components. However, certain modifications such as e.g., mercerization of lignocellulosic components result in obtaining a different polymorphic variety of cellulose. It has been difficult to determine the effect of different crystallographic structures of cellulose on the supermolecular structure of the polymer matrix and mechanical properties of the composites. Many attempts have been made to explain it by taking into account the nucleation abilities and hence the kinetics of crystallisation of the semicrystalline matrix in the presence of different varieties of cellulose or by the possible epitaxial growth of polymer chains on the surface of particular polymorphic varieties of cellulose.

Another problem widely discussed in the literature is the processing of thermoplastic polymer composites with lignocellulose fillers. Many authors have described a relation between the shearing forces occurring during processing and the supermolecular structure of the polymer matrix. This problem is well described in the processing of thermoplastic polymers but as far as the processing of composites is concerned, the appearance of certain polymorphic forms of the polymer matrix has not yet been fully explained. The difficulty is the quantitative description of shearing forces at the border of the polymer-filler phases. However, there is no doubt that to get a composite of the desired properties it is necessary to optimise the processing parameters. This paper also presents problems related to the effective recycling of the composites, viewed as a safe method for their utilisation.
